# Sol-Gel Synthesis and Photoluminescence Properties of a Far-Red Emitting Phosphor BaLaMgTaO_6_:Mn^4+^ for Plant Growth LEDs

**DOI:** 10.3390/ma16114029

**Published:** 2023-05-28

**Authors:** Niansi Fan, Quan Du, Rui Guo, Lan Luo, Li Wang

**Affiliations:** School of Physics and Materials Science, Nanchang University, 999 Xuefu Avenue, Honggutan New District, Nanchang 330031, China; fanns2023@163.com (N.F.);

**Keywords:** far-red emitting phosphor, BaLaMgTaO_6_:Mn^4+^, sol-gel method, photoluminescence, plant growth LEDs

## Abstract

Far-red (FR) emitting LEDs are known as a promising supplement light source for photo-morphogenesis of plants, in which FR emitting phosphors are indispensable components. However, mostly reported FR emitting phosphors are suffering from problems of wavelength mismatch with LED chips or low quantum efficiency, which are still far from practical applications. Here, a new efficient FR emitting double-perovskite phosphor BaLaMgTaO_6_:Mn^4+^ (BLMT:Mn^4+^) has been prepared by sol-gel method. The crystal structure, morphology and photoluminescence properties have been investigated in detail. BLMT:Mn^4+^ phosphor has two strong and wide excitation bands in the range of 250–600 nm, which matches well with a near-UV or blue chip. Under 365 nm or 460 nm excitation, BLMT:Mn^4+^ emits an intense FR light ranging from 650 to 780 nm with maximum emission at 704 nm due to ^2^E_g_ → ^4^A_2g_ forbidden transition of Mn^4+^ ion. The critical quenching concentration of Mn^4+^ in BLMT is 0.6 mol%, and its corresponding internal quantum efficiency is as high as 61%. Moreover, BLMT:Mn^4+^ phosphor has good thermal stability, with emission intensity at 423 K keeping 40% of the room temperature value. The LED devices fabricated with BLMT:Mn^4+^ sample exhibit bright FR emission, which greatly overlaps with the absorption curve of FR absorbing phytochrome, indicating that BLMT:Mn^4+^ is a promising FR emitting phosphor for plant growth LEDs.

## 1. Introduction

Like many other external environment conditions, such as temperature, water and minerals, light is essential for the growth and development of plants. Light provides an energy source for plant growth through photosynthesis and also serves as a light signal that modulates the entire process of plant growth and development [1,2,3]. Plants can mainly absorb blue (400–500 nm), deep-red (640–690 nm) and far-red (FR, 700–740 nm) lights [4,5,6]. Among them, FR light is indispensable for plant photo-morphogenesis, during which FR light absorbed by phytochrome leads to changes in plant florescence and growth [7,8]. Phytochromes have red light absorbing form (P_R_) and FR light absorbing form (P_FR_), and they can be transformed into each other depending on the wavelength of the irradiation light. P_R_ will convert to be P_FR_ under 660 nm deep-red light irradiation, and this process can be reversed under 730 nm FR light irradiation or nighttime conditions. The concentration of P_R_ and P_FR_ affects the physiological changes of plants, from vegetative to flowering growth. As a result, controlling the wavelength of irradiation light (deep-red or FR light) can change the concentration ratio of P_R_ to P_FR_ so as to regulate the flowering period of plants. Prolonged deep-red light irradiation can increase the concentration of P_FR_, resulting in early flowering of long-day plants. Raising the exposure time of FR light can increase the concentration of P_R_, which postpones the flowering time for long-day plants but brings forward the flowering time for short-day plants. In addition, FR light could also prevent bitter taste caused by excessive growth of vegetables and deterioration of food quality, which is very important for planting high-value vegetables. Therefore, by adjusting the spectral composition of light source, the growth and development process of plants could be regulated to improve the quality and yield of crops.

Traditional light devices, such as incandescent lamps and fluorescent lamps, have high energy consumption and low light efficiency, and generally they do not match the spectra required for plant growth. As a result, traditional light sources are not optimal for plant growth. Comparatively, light-emitting diodes (LEDs) show the extraordinary advantages of low energy consumption, high light efficiency, long lifetime, small volume and fast response time. Most importantly, the spectral composition of phosphor-converted LEDs (pc-LEDs) is tunable through optimizing the component and proportion of phosphors. Therefore, pc-LEDs have been offered as a promising light source for indoor plant cultivation and crop harvest [1,2,3,4,5,9].

Recently, Mn^4+^ ion as a rare-earth-free activator for red-emitting phosphors has attracted considerable attention [10,11,12,13,14,15]. With a 3d^3^ electron configuration and in different octahedral crystal field environments, Mn^4+^ ion exhibits strong and broad excitation band covering the region from NUV to visible, due to ^4^A_2g_ → ^4^T_1g_, ^2^T_2g_ and ^4^T_2g_ spin-allowed transitions. It can emit red/FR lights due to ^2^E_g_ → ^4^A_2g_ transition from 618 nm (13,840 cm^−1^) in KNaSnF_6_:Mn^4+^ [16] to 722 nm (13,840 cm^−1^) in SrTiO_3_:Mn^4+^ [17]. The emission wavelength of Mn^4+^ strongly depends on the covalence of Mn-ligand bonding [18]. At present, Mn^4+^-activated phosphors can be roughly classified as oxides and fluorides. The emission peaks of Mn^4+^ in fluorides are mainly located at shorter than 630 nm [19], while those in oxides are usually longer than 640 nm [20]. Therefore, it is feasible to search for an oxide host with a strong covalent effect in which Mn^4+^ can emit a FR light in line with the absorption of P_FR_ phytochrome. At present, there are a few reports [1,21,22,23] on FR emitting (700–740 nm) phosphors used for plant growth LEDs. However, most FR phosphors reported are still far from practical application due to their wavelength mismatch with chips, low quantum efficiency (QE) or other issues.

The double perovskite compound A_2_BXO_6_, possessing stable physical and chemical properties, can provide a perfect octahedral site [XO_6_] for Mn^4+^ incorporation, which shows excellent photoluminescence (PL) properties (e.g., La_2_ZnTiO_6_:Mn^4+^ [24], Ca_2_LaSbO_6_:Mn^4+^ [25], Ba_2_GdNbO_6_:Mn^4+^ [26] and Ca_2_LaTaO_6_:Mn^4+^ [27]). BaLaMgTaO_6_ (BLMT) [28] is also a double perovskite compound with [TaO_6_] octahedron structure, which is favorable for Mn^4+^ occupancy to exhibit excellent PL properties due to its ionic radius similar to Ta^5+^ (*r*(Mn^4+^) = 0.53 Å and *r*(Ta^5+^) = 0.64 Å when CN = 6). Recently, Zhou and co-workers [29] reported BLMT:Mn^4+^, Bi^2+^, Ca^2+^ FR emitting phosphor obtained by solid-state reaction method. However, the prepared phosphor sample has an irregular morphology of wide size distribution and serious agglomeration. Additionally, it shows poor thermal stability, with the PL intensity at 423 K reaching only 23% of the room temperature (RT) value.

In this study, BLMT:Mn^4+^ phosphor with uniform morphology, narrow size distribution and good dispersibility was synthesized using sol-gel method. The QE of BLMT:Mn^4+^ phosphor obtained by sol-gel route exceeds 60%. Its thermal stability is significantly improved with the PL intensity at 423 K maintaining close to 40% of the RT value. The crystal field analysis and nephelauxetic effect of Mn^4+^ in BLMT were also approximately evaluated. Eventually, the prototype LED devices were successfully fabricated by coating BLMT:Mn^4+^ phosphor on LED chips to verify its application in plant growth lighting.

## 2. Materials and Methods

### 2.1. Sample Preparation

A series of BLMT:*x*Mn^4+^ (*x* = 0.2, 0.4, 0.6, 0.8, 1.0 and 1.2 mol%) polycrystalline powders were prepared by sol-gel method. All raw materials MgCO_3_ (99%, Macklin), BaCO_3_ (99%, Macklin), La_2_O_3_ (99.9%, Macklin), MnCO_3_ (99.9%, Macklin), TaCl_5_ (99.9%, Macklin), can be used directly without purification. According to the stoichiometric ratio of BaLaMgTa_1−*x*_O_6_:*x*Mn^4+^, MnCO_3_, MgCO_3_, BaCO_3_ and La_2_O_3_ were dissolved in dilute nitric acid (HNO_3_, AR, Sinopharm, Beijing, China) to form a nitrate solution and TaCl_5_ was dissolved in ethanol with addition of ammonia (NH_3_·H_2_O, AR, Sinopharm) to obtain white sediment. After several rounds of cleaning with deionized water, the white sediment was mixed with the above nitrate solution under vigorous stirring. Then, the appropriate amount of citric acid and ethylene glycol were added as a complexing agent and stabilizer. In this work, the molar ratio of citric acid to metallic ions and ethylene glycol was 1:1:1. Finally, the appropriate amount of diluted ammonia water was dropped into the solution to keep its pH value at 7–8. After continuous stirring for 4 h, a uniform and transparent sol were formed. The obtained sol was dried in an oven at 120 °C for 8 h and then transformed into a light brown xerogel. Ground into powder, it was pre-calcined at 500 °C for 5 h and calcined at 1450 °C for 10 h. When cooled down to RT and ground again, BLMT:Mn^4+^ fine powder samples were ultimately achieved.

The LED devices were fabricated combining as-prepared BLMT:Mn^4+^ phosphor with LED chips. According to the weight ratio of 1:1, the phosphor and epoxy resin were fully blended and then coated onto the NUV (~365 nm) and blue InGaN (~460 nm) chips, respectively. After they solidified at 80 °C for 1.5 h, the LED devices for plant growth lighting were obtained.

### 2.2. Characterization

The thermal analysis was conducted on a simultaneous thermal analyzer (NETZSCH STA 449 F5) at a heating rate of 10 °C/min within the temperature range of RT—1400 °C. The X-ray diffraction (XRD) patterns data were obtained by a X-ray diffractometer (SmartLab-9KW, Rigaku Corporation, Tokyo, Japan) using Cu Kα radiation (λ = 1.5405 Å) with a scanning step of 0.02° at 40 kV and 40 mA. The morphology observation and element analysis were carried out by a field-emission scanning electron microscopy (FE-SEM, Quanta 200FEG, FEI Company, Hillsboro, USA) equipped with an Aztec X-Max 80 energy-dispersive X-ray spectrometer (EDS). The PL and PL excitation (PLE) spectra, decay curves and internal QE (IQE) were measured using a spectrometer (FS15, Edinburgh Instruments, Livingston, UK) with a 150-W Xe lamp as the excitation source and a barium sulfate-coated integrating sphere. The temperature-dependent PL spectra were measured in the range of 298–498 K by a steady-state spectrofluorometer (FLS1000, Edinburgh Instruments, Livingston, UK) with a pulse diode laser as the excitation source and a temperature controlling system. The electroluminescence (EL) spectrum and Commission International de I’Eclairage (CIE) chromaticity coordinates of LEDs were measured by a fast spectroradiometer (HAAS-2000, Everfine, Hangzhou, China). All the measurements were conducted at room temperature except for the thermal analysis and the temperature-dependent PL spectra.

## 3. Results and Discussion

### 3.1. Structure and Morphology

**Figure 1:**
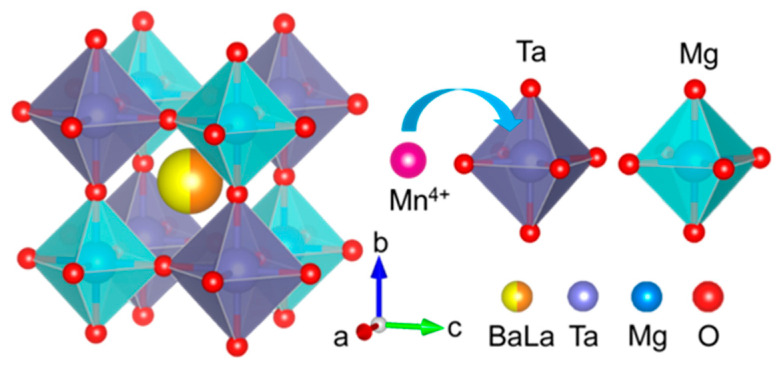
Crystal structure of BLMT unit cell and coordination polyhedrons of Ta^5+^ and Mg^2+^.

Figure 1 presents the crystal structure of BLMT unit cell depicted according to the Inorganic Crystal Structure Database (ICSD#160281). BLMT crystalizes in a double perovskite structure with a space group of $Fm\bar{3}m$. Its lattice parameters are *a* = 8.06632 Å, *V* = 524.84 Å^3^ and *Z* = 4. In the crystal structure of BLMT, [MgO_6_] and [TaO_6_] octahedrons alternatively connect to each other by sharing vertexes to form a 3D network structure, while Ba and La atoms randomly occupy the structure interstices which are surrounded by 12 oxygen atoms. In view of the similar ionic radius in the same coordination environment, Mn^4+^ (CN = 6, *r* = 0.53 Å) ions preferentially replace Ta^5+^ (CN = 6, *r* = 0.64 Å) ion sites in BLMT lattice.

**Figure 2:**
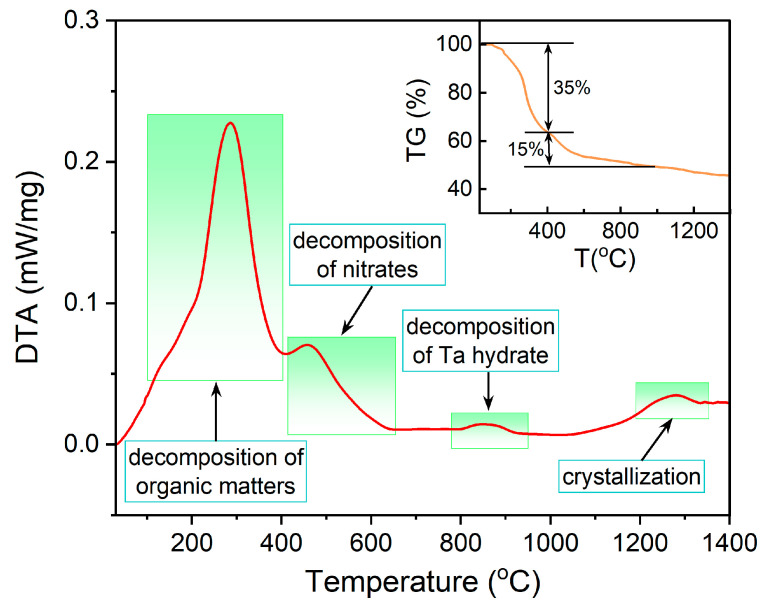
DTA-TG profile of BLMT:0.6%Mn^4+^ precursor xerogel.

In order to better understand the thermal decomposition and crystallization process, Figure 2 provides the DTA-TG curves of BLMT:0.6%Mn^4+^ precursor xerogel in a RM-1400 °C range. There are obviously four exothermic peaks in the DTA profile at <400, 400–650, 800–950 and 1200–1300 °C, respectively. Below 400 °C, the DTA curve presents a broad exothermic peak accompanied by a large drop with a mass loss of about 35% in the TG curve, which is mainly caused by the decomposition of organic matters and the evaporation of residual water [30,31]. The exothermic peaks at 400–650 °C and 800–950 °C result from the decomposition of metal nitrates [32,33,34] and tantalum precipitate [35], respectively. The total mass loss of the decomposition of metal nitrates and tantalum precipitate is approximately 15%. When the temperature rises to 1200 °C, the sample starts to crystallize and its exothermic peak temperature is about 1275 °C.

**Figure 3:**
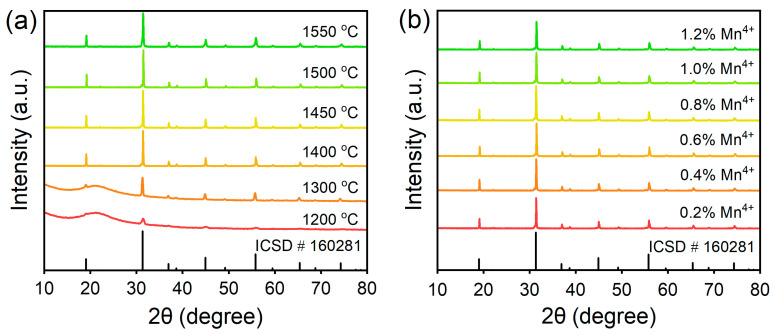
XRD patterns of (**a**) BLMT:Mn^4+^ samples synthesized at different sintering temperatures; (**b**) BLMT:Mn^4+^ samples with different concentration synthesized at 1450 °C.

The structure and luminescence properties of phosphors are greatly affected by the sintering temperature. To determine the optimal sintering temperature, BLMT:Mn^4+^ samples with Mn^4+^ concentration of *x* = 0.6% were synthesized at 1200, 1300, 1400, 1450, 1500 and 1550 °C, respectively. The XRD patterns of BLMT:0.6%Mn^4+^ samples at different sintering temperatures and the standard pattern of BLMT (ICSD#160281) are shown in Figure 3a. It can be seen from Figure 3a that the samples fired at different temperature exhibit different crystalline properties. The XRD patterns of BLMT:0.6%Mn^4+^ samples are consistent with ICSD#160281, which indicates BLMT:Mn^4+^ samples have the same crystal structure as the BLMT compound. In addition, the samples calcined at 1200 °C and 1300 °C present typical broad diffraction bands of amorphous phase materials at low angles. It can be inferred that the samples start to crystallize but the crystallization is not complete at temperatures below 1400 °C. When the sintering temperature is equal to or higher than 1400 °C, the samples have better crystallinity with no detected impurity. This is in agreement with the thermal analysis result shown in Figure 2. Considering the effects of sintering temperature on particle morphology (see in Figure S1) and PL intensity (see in Figure S2) of samples, as well as the factor of energy consumption, it is determined that the optimal sintering temperature is 1450 °C.

Figure 3b displays the XRD patterns of BLMT:*x*Mn^4+^ (*x* = 0.2, 0.4, 0.6, 0.8, 1.0 and 1.2 mol%) prepared at 1450 °C. It is can be seen that the XRD patterns of BLMT:*x*Mn^4+^ samples are well conformed with the standard data of BLMT (ICSD# 160281) and no impurity peaks are observed. This indicates that the introduction of Mn^4+^ ion does not cause significant change in the crystal structure of BLMT within the above doping concentration range, and Mn^4+^ ions have been effectively incorporated into the host lattice.

**Figure 4:**
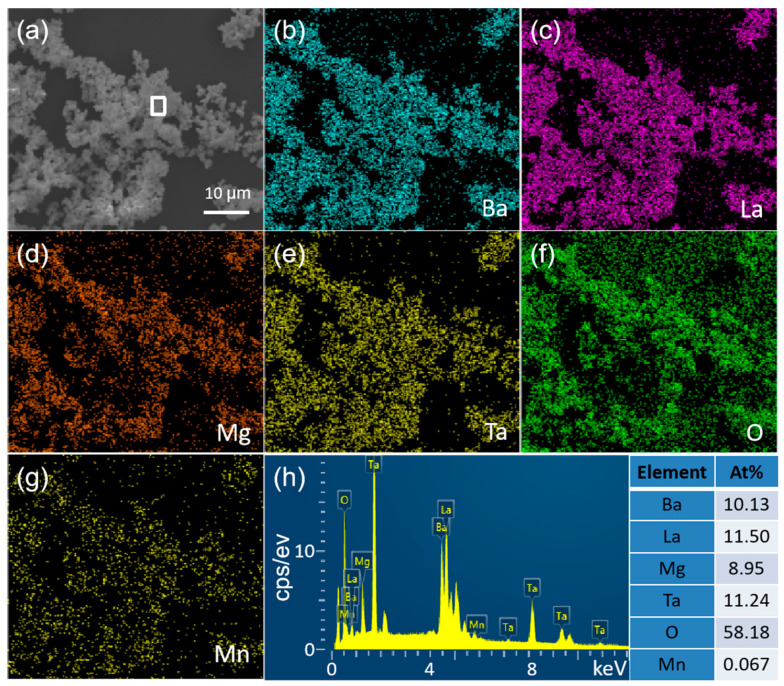
(**a**) SEM image of BLMT:0.6%Mn^4+^ sample and (**b**–**g**) its corresponding element mappings; (**h**) EDS spectrum and element composition for BLMT:0.6%Mn^4+^ sample.

The particle size and morphology of phosphors have great influence on their luminescence properties. The SEM image of typical BLMT:0.6%Mn^4+^ phosphor prepared at 1450 °C and its element mapping is shown in Figure 4a. As can be seen in Figure 4a, the BLMT:0.6%Mn^4+^ sample exhibits smooth surface, good dispersivity and uniform morphology with appropriate size of 1–3 μm, and homogeneous distribution of Ba, La, Ta, Mg, Mn and O elements in the whole observing field. Furthermore, with an increase in temperature, the particle size grows almost monotonously from 0.5 to 4 μm and the particle surface becomes smooth (see Figure S1). When the temperature is higher than 1450 °C, BLMT:Mn^4+^ particles show serious agglomeration and irregular shape. Figure 4b provides the EDS spectrum and element composition of the selected zone. EDS spectrum presents the peaks of Ba, La, Mg, Ta, O and Mn elements, and the atom ratio is calculated to be 10.13:11.50:8.95:11.24:58.18:0.067, which is much closer to the theoretical composition of BaLaMgTa_0.994_O_6_:0.006Mn^4+^.

### 3.2. PL Properties

**Figure 5:**
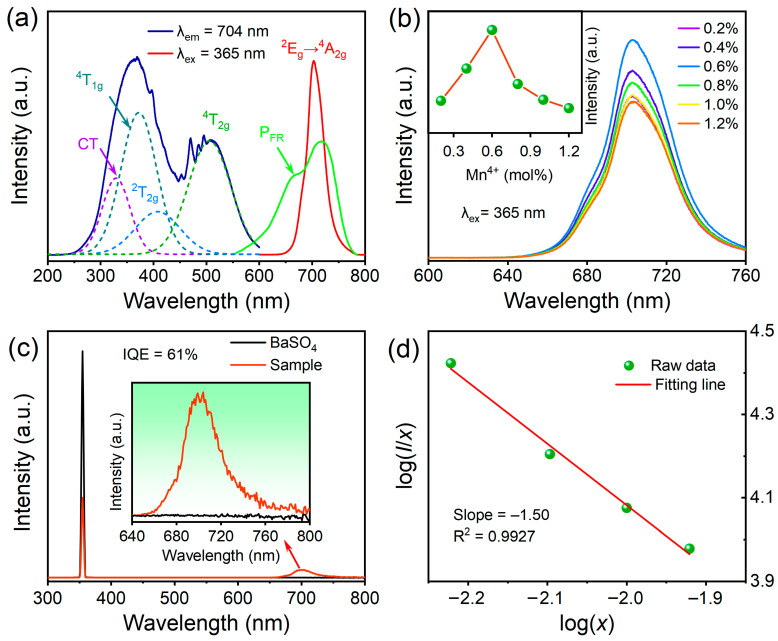
(**a**) PLE (*λ*_em_ = 704 nm) and PL (*λ*_ex_ = 365 nm) spectra of BLMT:0.6%Mn^4+^; (**b**) PL spectra of BLMT:*x*Mn^4+^ (*x* = 0.2–1.2%) under 365 nm excitation, Inset: concentration-dependence of PL intensity of BLMT:*x*Mn^4+^; (**c**) Excitation line of BaSO_4_ and PL spectrum of BLMT:0.6%Mn^4+^ collected using an integrating sphere; (**d**) Linear relationship of log(*I*/*x*) versus log(*x*) for BLMT:Mn^4+^.

In order to understand the luminescence of BLMT:Mn^4+^ phosphor, the PL and PLE spectra of the as-prepared samples were recorded at RT. As illustrated in Figure 5a, the PLE spectrum contains two strong and broad excitation bands in the wavelength range from 250 to 600 nm. The stronger band peaks at 365 nm, which matches the commercial NUV LED chip. By Gaussian fitting, the PLE spectrum is divided into four Gaussian bands peaking at 329 nm (30,395 cm^−1^), 372 nm (26,882 cm^−1^), 408 nm (24,510 cm^−1^) and 506 nm (19,763 cm^−1^), which corresponds to the Mn^4+^-O^2−^ charge transfer (CT) and the spin-allowed transitions of Mn^4+^ ion from the ground state ^4^A_2g_ to the excited states ^4^T_1g_, ^2^T_2g_ and ^4^T_2g_, respectively. Under the excitation at 365 nm, BLMT:Mn^4+^ phosphor exhibits an intense and broad FR emission centered at 704 nm due to the ^2^E_g_→^4^A_2g_ forbidden transition of Mn^4+^ ion. The full width at half maxima (FWHM) of the emission band is 36 nm, which largely overlaps with the absorption curve of phytochrome P_FR_. It is noted that BLMT:Mn^4+^ phosphor also exhibits a strong excitation intensity in blue region (as seen in Figure 5a), which is also suitable to be excited by a blue chip. Under the excitation of 460 nm, BLMT:Mn^4+^ phosphor also shows a strong and wide FR emission in the range of 650–800 nm (see in Figure S3). Therefore, it can be inferred that BLMT:Mn^4+^ phosphor has great application potential in plant growth LED devices.

Figure 5b shows the PL spectra of BLMT:*x*Mn^4+^ (*x* = 0.2–1.2%) samples under 365 nm excitation. The shape and peak positions of all PL spectra are almost identical to each other except for the emission intensity, which increases initially with the increase of Mn^4+^ concentration and then declines after reaching the peak value at *x* = 0.6 mol%. This is because the concentration quenching effect occurs at high Mn^4+^ content. The primary mechanism of the concentration quenching may be related to the critical distance (*R*_c_) of the activators, which could be estimated by the formula [36,37] below:

$$R_{c}\approx 2{\left[\frac{3V}{4\pi X_{c}N}\right]}^{\frac{1}{3}}$$

where *V* is the cell volume of the host lattice, *X*_c_ is the critical concentration of the activated ion and *N* is the number of available sites for the dopant in one unit cell. When *R*_c_ is less than 5 Å, exchange interaction is the main mechanism of concentration quenching. In the case of BLMT:Mn^4+^, the values of *V*, *X*_c_ and *N* are *V* = 524.84 Å^3^, *X*_c_ = 0.006 and *N* = 4, respectively, and thus the calculated value of *R*_c_ is about 34.7 Å, which is much larger than the maximum distance required for exchange interaction (5 Å). This indicates that the concentration quenching phenomenon is mainly attributed to the electric multipolar interactions rather than the exchange interaction, which can be elucidated by the following formula:

$$\log\left(\frac{I}{x}\right)=-\frac{\theta }{3}\log x+A$$

where *I* is the PL intensity at a given dopant concentration *x* and A is a constant. *θ* = 6, 8 and 10 correspond to the electric dipole–dipole, dipole–quadrupole and quadrupole–quadrupole interaction, respectively [38]. By linear fitting the relationship between log(*I*/*x*) and log(*x*), the slope of fitting line is −*θ*/3 = −1.50 (see Figure 5d). As a result, the value *θ* = 4.50 is close to 6, which indicates that the concentration quenching mechanism could be dominated by the dipole–dipole interaction in BLMT:Mn^4+^.

QE, a vital parameter for luminescence materials, is defined as the number ratio of the emitted to absorbed photons. The IQE (*η*_int_) of BLMT:0.6%Mn^4+^ phosphor is calculated by use of the following equation [39]:

$$\eta_{\mathrm{int}}=\frac{\int L_{S}}{\int E_{R}-\int E_{S}}$$

where *E*_S_ and *E*_R_ refer to the spectra of excitation light with and without sample in the integrating sphere, respectively, and *L*_S_ is the emission spectrum of the sample. Based on the recorded excitation and emission spectra, the IQE of the sample is determined to be 61% (see Figure 5c), which is comparable to that reported in [29], and much higher than many other Mn^4+^-activated double perovskite phosphor reported before, such as Ca_2_LaSbO_6_ (IQE: 52%) [25], Ca_2_LaTaO_6_ (IQE: 35%) [27], Sr_2_GdNbO_6_ (IQE: 37%) [40], Gd_2_ZnTiO_6_ (IQE: 40%) [41] and CaLaMgTaO_6_ (IQE: 28%) [42].

**Figure 6:**
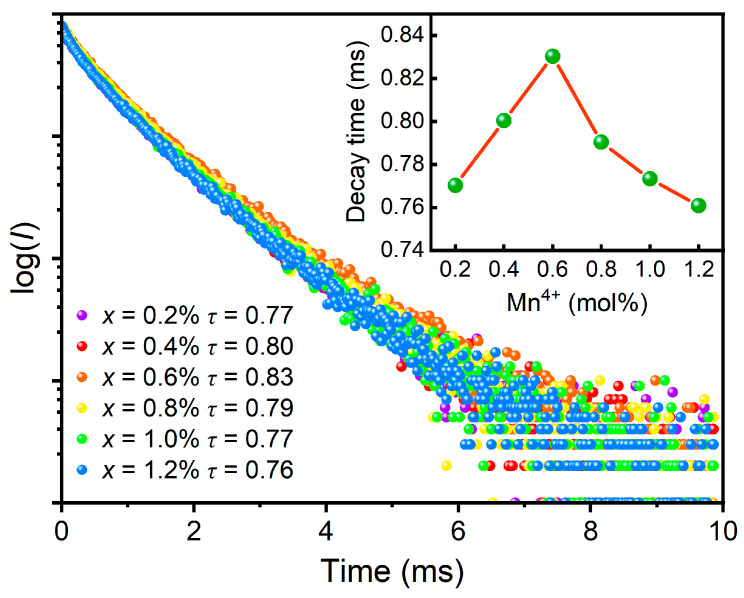
Decay curves (*λ*_ex_ = 365 nm, *λ*_em_ = 704 nm) of BLMT:*x*Mn^4+^ (*x* = 0.2–1.2%), Inset: dependence of luminescence decay time on Mn^4+^ concentration.

To understand the process of luminescence kinetics in detail, Figure 6 presents the PL decay curves (*λ*_ex_ = 365 nm, *λ*_em_ = 704 nm) of the BLMT:*x*Mn^4+^ (*x* = 0.2–1.2%) samples at RT. All the decay curves can be well fitted with the second-order exponential function, as expressed below:

$$I\left(t\right)=I_{0}+A_{1}\exp\left(-t/\tau_{1}\right)+A_{2}\exp\left(-t/\tau_{2}\right)$$

where *I*(*t*) is the fluorescence intensity at time *t* and *I*_0_ is the initial intensity. A_1_ and A_2_ are constants and *τ*_1_ and *τ*_2_ correspond to the decay time for the exponential components. The average lifetime *τ* can be calculated via the following equation:

$$\tau =\left(A_{1}\tau_{1}^{2}+A_{2}\tau_{2}^{2}\right)/\left(A_{1}\tau_{1}+A_{2}\tau_{2}\right)$$

The calculated values of average lifetime for BLMT:*x*Mn^4+^ (*x* = 0.2–1.2%) samples are 0.77, 0.80, 0.83, 0.79, 0.77 and 0.76 ms, respectively. As reported in previous research [43], the results of lifetime value are in the millisecond scale which is due to the forbidden characteristics of the 3D-shell transitions in Mn^4+^ ion. As shown in the inset of Figure 6, the decay lifetime first increases and then decreases with the increase of Mn^4+^ content, and the maximum appears at *x* = 0.6%, which is in agreement with the concentration dependence of PL intensity. This is because the energy migration among the Mn^4+^ ions at high concentration leads to a high nonradiative transition probability.

### 3.3. Crystal Field Analysis

**Figure 7:**
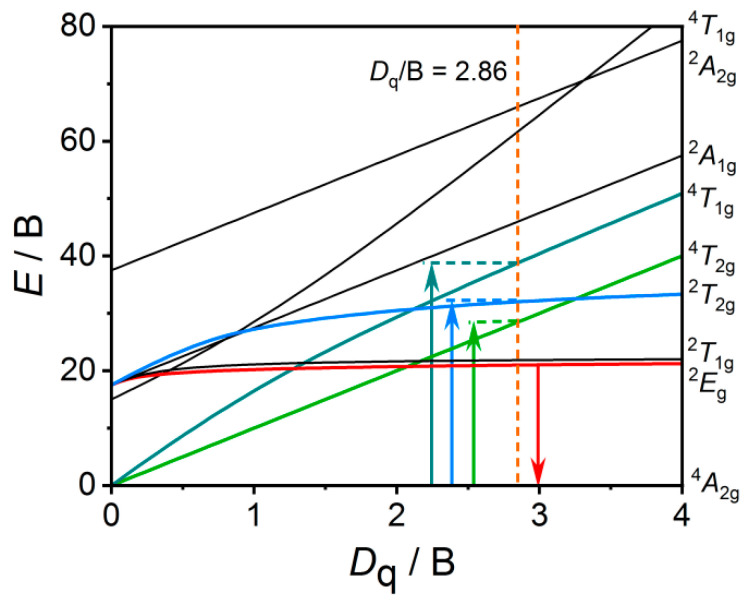
Tanabe-Sugano energy level diagram for Mn^4+^ in octahedral crystal field of BLMT host.

The luminescence mechanism of Mn^4+^ ion in octahedron environment may be explained by using Tanabe-Sugano energy level diagram, as presented in Figure 7. After absorbing enough energy, some electrons at ground state ^4^A_2g_ will transit to the excited state ^4^T_1g_, ^4^T_2g_ or ^2^T_2g_, then relax to the lower excited state ^2^E_g_ via nonradiative process, and finally return to the ground state with a red or FR emission. The emission of Mn^4+^ is always sensitive to the crystal field environment in host. The local crystal field strength (*D*_q_) can be evaluated by the energy gap between levels ^4^A_2g_ and ^4^T_2g_ according to the following formula [44,45]:

*D*_q_ = *E*(^4^A_2g_ → ^4^T_2g_)/10

The calculated *D*_q_ and the energy difference between *E*(^4^A_2g_ → ^4^T_1g_) and *E*(^4^A_2g_ → ^4^T_2g_) can be used to estimate the Racah parameter B through the following equation [40]:

$$\frac{D_{q}}{B}=\frac{15\left(\alpha -8\right)}{\left(\alpha^{2}-10\alpha \right)}$$

where the parameter *α* is expressed as:

α=E(A42g→T41g)−EA42g→T42gDq

Based on the peak energy of ^2^E_g_ → ^4^A_2g_ transition, another Racah parameter C can be calculated according to the formula below [18]:

EE2g→A42gB=3.05CB−1.8BDq+7.9

In light of the PL spectra of BLMT:Mn^4+^ phosphor (see Figure 5a), the values of *E*(^4^A_2g_ → ^4^T_1g_), *E*(^4^A_2g_ → ^4^T_2g_) and *E*(^2^E_g_ → ^4^A_2g_) are determined to be 26,882 cm^−1^ (372 nm), 19,763 cm^−1^ (506 nm) and 14,205 cm^−1^ (704 nm), respectively. Therefore, the crystal field parameters *D*_q_, B and C calculated according to the above formulas are 1976 cm^−1^, 690 cm^−1^ and 3012 cm^−1^, respectively. In general, the crystal field will be regarded as the strong one when the value of *D*_q_/B is larger than 2.2. In the case of BLMT:Mn^4+^, the *D*_q_/B value can be calculated to be 2.86, which suggests that Mn^4+^ experiences strong crystal field in BLMT crystal.

According to the Tanabe-Sugano energy diagram (as seen in Figure 7), the *E*/B curve of ^2^E_g_ is almost a horizontal line which is barely affected by the crystal field strength. In fact, the emission energy of Mn^4+^ depends on the nephelauxetic effect relating to the covalence of the Mn-ligand bond, and thus a ratio (*β*_1_) was introduced to reflect the repulsion between the electronic pairs and ligands, which can be estimated through the following equation [18]:

$$\beta_{1}=\sqrt{{\left(\frac{B}{B_{0}}\right)}^{2}+{\left(\frac{C}{C_{0}}\right)}^{2}}$$

where B_0_ = 1160 cm^−1^ and C_0_ = 4303 cm^−1^ are the Racah parameters of the free Mn^4+^ ions. As a result, the value of *β*_1_ is calculated to be 0.9186 for BLMT:Mn^4+^. According to Brik [18], the energies of the ^2^E_g_ → ^4^A_2g_ transition for most Mn^4+^-activated phosphors can be estimated by the following linear equation:

*E*(^2^E_g_ → ^4^A_2g_) = −880.49 + 16261.92*β*_1_ ± *σ*

where *σ* is the root-mean square deviation with the value of 332 cm^−1^. Thus, the value of *E*(^2^E_g_ → ^4^A_2g_) for BLMT:Mn^4+^ phosphor should be in the range of 13,725–14,389 cm^−1^. As determined above, the experimental value of *E*(^2^E_g_ → ^4^A_2g_) of Mn^4+^ in BLMT:Mn^4+^ phosphor is 14,205 cm^−1^, which indicates that the crystal field analysis results are credible.

### 3.4. Thermal Stability

**Figure 8:**
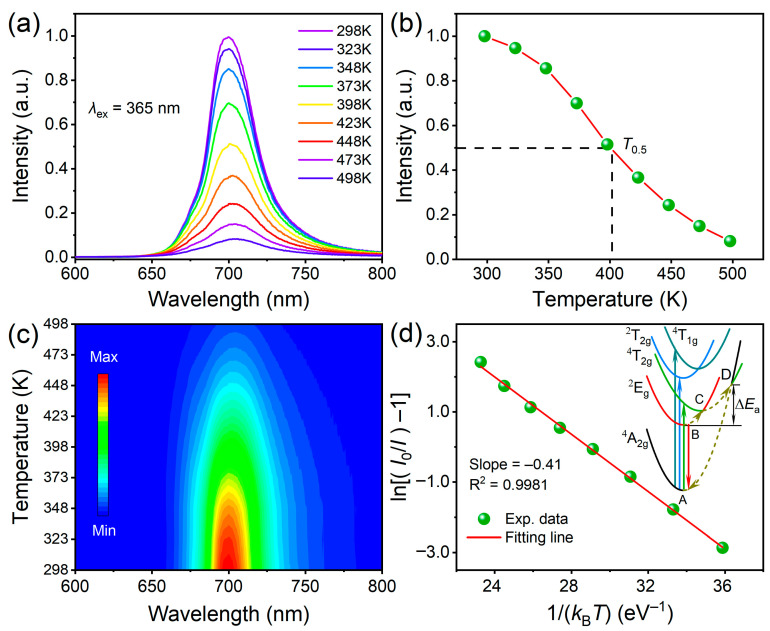
(**a**) Temperature-dependent PL spectra of BLMT:Mn^4+^ ranging from 298 to 498 K; (**b**) Temperature dependence of the integrated PL intensity; (**c**) The 2D plot of emission dependence on temperature; (**d**) Relationship between ln[(*I*_0_/*I*) − 1] versus 1/(*k*_B_*T*), Inset: thermal quenching mechanism diagram for Mn^4+^ ion in BLMT.

Generally, the temperature of LEDs can climb up to 423 K when they are in working state. Therefore, for phosphors used in LED devices, their thermal stability is critical since the temperature has a great influence on the luminescence performances [46,47]. On this account, the temperature-dependent PL spectra of BLMT:Mn^4+^ phosphor were recorded within the temperature range of 298–498 K with an interval of 25 K, as depicted in Figure 8a. As seen in Figure 8a, all PL spectra have the same profile but the PL intensity, which decreases monotonously with the increase of temperature attributed to the thermal quenching effect, as seen in Figure 8b. The quenching temperature *T*_50_, a primary parameter for phosphors applied to LEDs devices, is defined as the temperature at which the PL intensity reaches 50% of the original value. The value of *T*_50_ is higher than 400 K for BLMT:Mn^4+^ phosphor, and the PL intensity at 423 K remains near 40% of the value at RT, which is much higher than the 23% reported in [29], indicating that BLMT:Mn^4+^ phosphor prepared by sol-gel method has better thermal stability. To exhibit the PL spectra evolution with temperature more clearly, Figure 8c presents the 2D plot of the temperature-dependent PL spectra of BLMT:Mn^4+^ phosphor.

The thermal quenching behavior of phosphors is a nonradiative relaxation related to temperature, which can be illustrated by the coordination energy diagram, as shown in the inset of Figure 8d. At RT, the electrons of Mn^4+^ are transported from the ground state ^4^A_2g_ to the excited state ^4^T_2g_ or ^4^T_1g_ under UV excitation, and then relax to the lowest excited state ^2^E_g_ in a nonradiative way. Finally, the electrons at excited state ^2^E_g_ return to the ground state ^4^A_2g_ with an emission at ~700 nm. However, when the temperature rises, part of the electrons situated in ^2^E_g_ excited state get enough energy Δ*E*_a_ and jump to the crossover point D of ^4^T_2g_ and ^4^A_2g_ parabolas along B-C-D path, and eventually relax to the ground state ^4^A_2g_ with no light emission. Δ*E*_a_, known as the thermal activation energy, is the energy difference between the bottom point B of ^2^E_g_ parabola and the crossover point D, which can be determined by following equation [48]:

$$I=\frac{I_{0}}{1+\mathrm{Aexp}\left(-\Delta E_{a}/k_{B}T\right)}$$

where *I* and *I*_0_ are the PL intensity at given temperature *T* and initial temperature *T*_0_, respectively. A is a constant and *k*_B_ is the Boltzmann constant (8.629 × 10^−5^ eV·K^−1^). By a linear fitting of ln[(*I*_0_/*I*) − 1] and 1/(*k*_B_*T*), the slope (−Δ*E*_a_) can be obtained. As shown in Figure 8d, the slope of fitting line is −0.41, so the thermal activation energy is Δ*E*_a_ = 0.41 eV for BLMT:Mn^4+^ phosphor, which is larger than other Mn^4+^ activated double perovskite phosphors, such as La_2_ZnTiO_6_:Mn^4+^ (0.30 eV) [24], Ca_2_LaSbO_6_:Mn^4+^ (0.36 eV) [25], Ba_2_GdNbO_6_:Mn^4+^ (0.36 eV) [26], Ca_2_LaTaO_6_:Mn^4+^ (0.32 eV) [27], Ca_2_LuTaO_6_:Mn^4+^ (0.25 eV) [49] and SrLaZnTaO_6_:Mn^4+^ (0.34 eV) [50].

### 3.5. EL Properties of Fabricated LED Device

**Figure 9:**
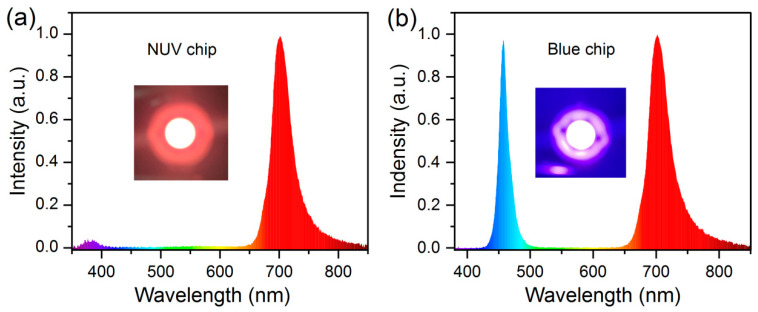
EL spectrum of the fabricated LED driven at 3 V and 100 mA based on (**a**) NUV and (**b**) blue chips.

In order to further evaluate the application potential of BLMT:0.6%Mn^4+^ phosphor in plant growth lighting, the LED devices were fabricated by coating BLMT:0.6%Mn^4+^ phosphor samples on NUV (~365 nm) and blue (~460 nm) chips. Figure 9 shows the electroluminescence (EL) spectra of as-fabricated LED devices driven at 3 V and 100 mA. Both EL spectra present an intense FR emission in the range of 650–850 nm, identical to the PL spectrum of BLMT:Mn^4+^ phosphor (see in Figure 5a), which is requisite light for plant growth. Additionally, there are a weak emission band in range of 350–400 nm due to NUV chip (Figure 9a) and another intense emission band peaking at 460 nm from the blue chip (Figure 9b), and the latter can also be used for plant growth lighting. The insets of Figure 9a,b exhibit bright lights emitted from as-assembled LED lamps. Moreover, driven at different currents, the LED devices show perfect stability in EL spectra (see Figure S4). All results demonstrate that BLMT:Mn^4+^ FR emitting phosphor has great application potential in plant growth lighting.

## 4. Conclusions

A FR emitting double-perovskite phosphor BLMT:Mn^4+^ was successfully synthesized by sol-gel method. BLMT:Mn^4+^ phosphor shows two strong and broad excitation bands in NUV and blue regions, and when excited by 365 nm or 460 nm light, it produces an intense FR emission peaking at 704 nm owing to the ^2^E_g_ → ^4^A_2g_ transition of Mn^4+^. The FWHM of FR emission is as wide as nearly 40 nm, which overlaps greatly with the absorption spectrum of P_FR_ phytochrome. When the Mn^4+^ concentration is 0.6 mol%, the PL QE of BLMT:Mn^4+^ is as high as more than 60%. BLMT:Mn^4+^ phosphor has a good thermal stability with an activation energy of 0.41 eV. According to crystal field analysis, the parameters *D*_q_, B and C calculated are 1976 cm^−1^, 690 cm^−1^ and 3012 cm^−1^, respectively, and the *D*_q_/B value is 2.86, which suggests that Mn^4+^ experiences strong crystal field in BLMT crystal. The FR LED devices constructed by combining NUV/blue chips with the as-prepared BLMT:Mn^4+^ samples demonstrate that BLMT:Mn^4+^ is a promising FR emitting phosphor for plant growth LEDs.

## Data Availability

The data supporting the findings of this study are available within the article and its Supplementary Materials.

## Supplementary Materials

The following supporting information can be downloaded at: https://www.mdpi.com/article/10.3390/ma16114029/s1, Figure S1: SEM images of BLMT:Mn^4+^ samples prepared at (a) 1200 °C, (b) 1300 °C, (c) 1400 °C, (d) 1450 °C, (e) 1500 °C, and (f) 1550 °C; Figure S2: (a) PL spectra of BLMT:Mn^4+^ samples prepared at temperature of 1200–1550 °C, Inset: temperature dependence of PL intensity for BLMT:Mn^4+^ samples; Figure S3: PL spectra of BLMT:Mn^4+^ samples excited by 365 nm and 460 nm, respectively; Figure S4: EL spectra of as-assembled LED lamps driven by different currents based on (a) NUV and (b) blue chips.
