# Supplementary material for: Sol-Gel Synthesis and Photoluminescence Properties of a Far-Red Emitting Phosphor BaLaMgTaO6:Mn4+ for Plant Growth LEDs
Source: Materials (Basel). 2023 May 28;16(11):4029. doi: 10.3390/ma16114029 (PMC10254883; doi:10.3390/ma16114029)
Supplement: Supplementary file 1 [file materials-16-04029-s001.zip › materials-2406444-supplementary.pdf]

Supplementary Materials

Sol-Gel Synthesis and Photoluminescence Properties of a Far-Red Emitting Phosphor $BaLaMgTaO_6:Mn^{4+}$ for Plant Growth LEDs

Fan †, Quan Du †, Rui Guo *, Lan Luo and Li Wang

School of Physics and Materials Science, Nanchang University, 999 Xuefu Avenue, Honggutan New District, Nanchang 330031, China

* Correspondence: ruig_711@hotmail.com

† These authors contributed equally to this work.

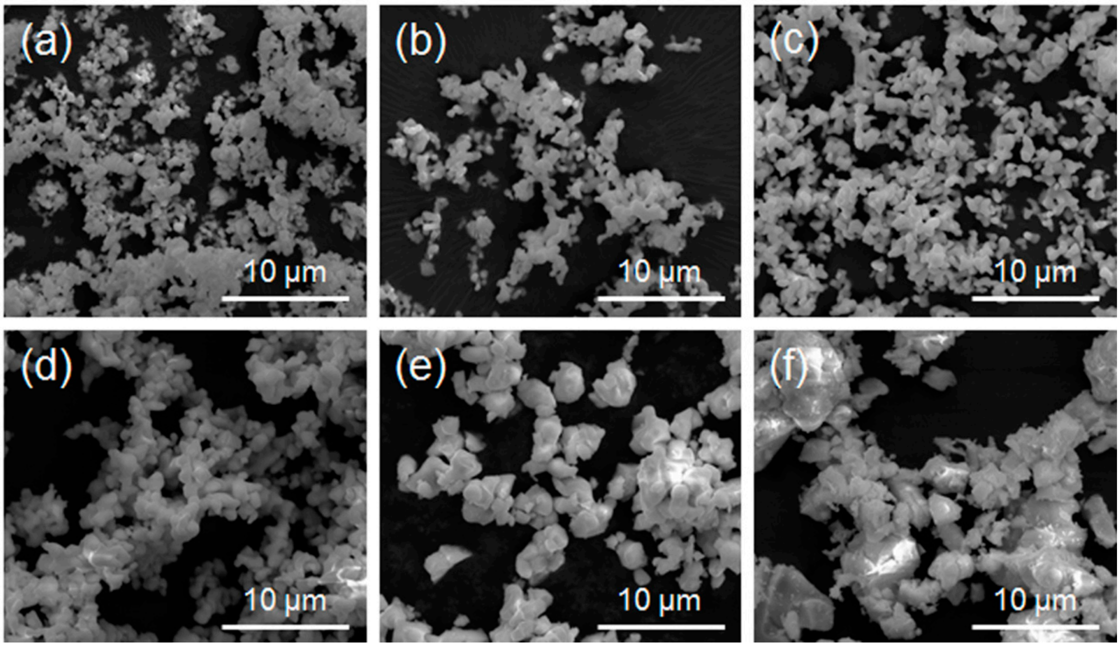

Figure S1. SEM images of BLMT:Mn^{4+} samples prepared at (a) 1200 ºC, (b) 1300 ºC, (c) 1400 ºC, (d) 1450 ºC, (e) 1500 ºC, and (f) 1550 ºC. With the increase of temperature,

the particle size grows almost monotonously from 0.5 to 4 μm, and the particle surface becomes smooth. When the temperature is higher than 1450 ºC, BLMT:Mn^{4+} particles show serious agglomeration and irregular shape.

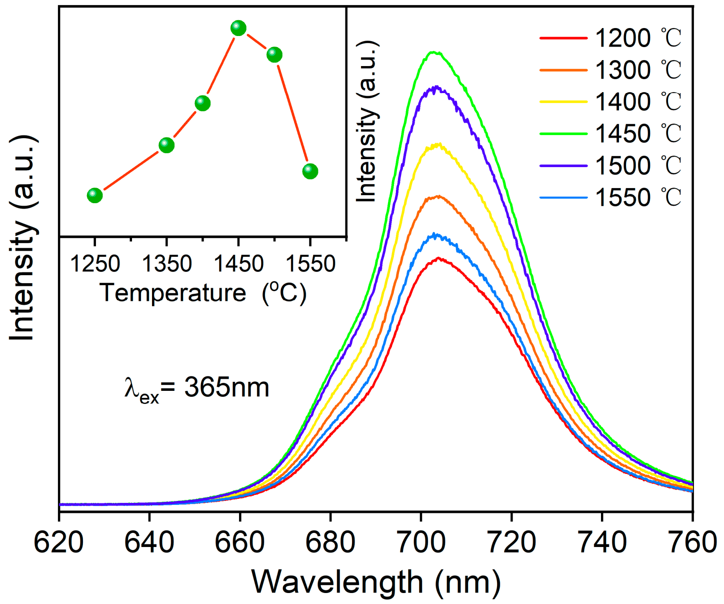

Figure S2. (a) PL spectra of BLMT:Mn^{4+} samples prepared at temperature of 1200-1550 ºC, Inset: temperature dependence of PL intensity for BLMT:Mn^{4+} samples. At all

sintering temperature, the shape and peak position of all PL spectra are almost identical to each other except for the emission intensity, which increases initially with the increase of temperature, and then declines after reaching the peak value at $T = 1450$ °C.

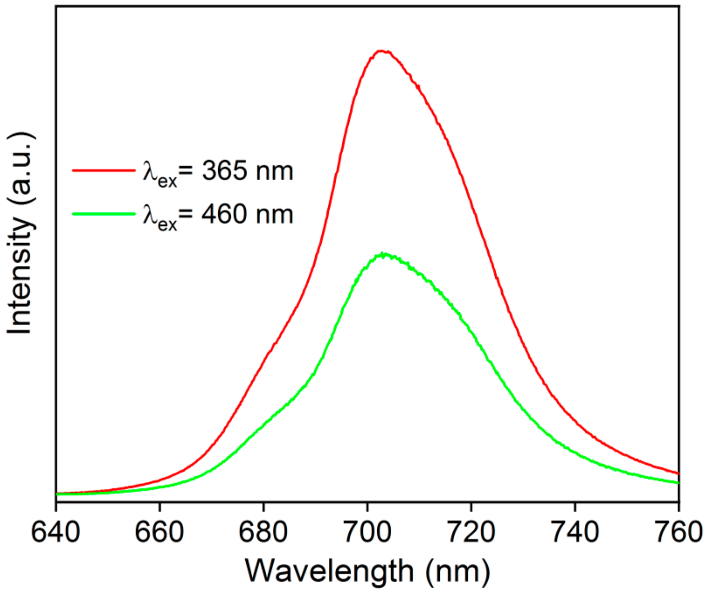

Figure S3. PL spectra of BLMT:Mn^{4+} samples excited by 365 nm and 460 nm, respectively. Fig S3 shows that BLMT:Mn^{4+} emits a intense FR light excited by both 365 and 460 nm lights.

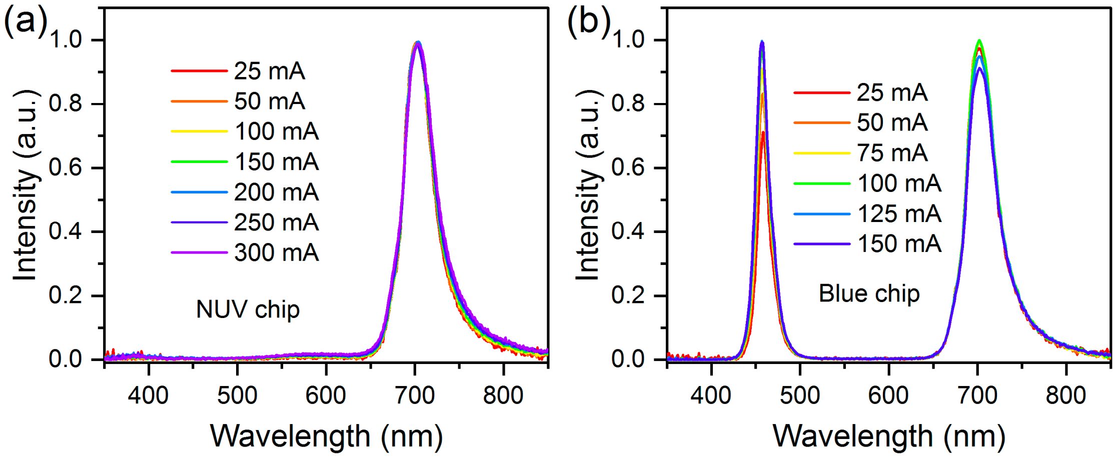

Figure S4. EL spectra of as-assembled LED lamps driven by different currents based on (a) NUV and (b) blue chips. Driven at different currents, the LED devices show perfect stability in EL spectra, which indicates that BLMT:Mn^{4+} FR emitting phosphor has great application potential in plant growth lighting.
